# Congenital lipoid adrenal hyperplasia in twin sisters

**DOI:** 10.1186/1687-9856-2013-S1-P113

**Published:** 2013-10-03

**Authors:** Hye Won Park, Byung Ok Kwak, Han-Wook Yoo, Sochung Chung

**Affiliations:** 1 Department of Pediatrics, Konkuk University Medical Center, Konkuk University School of Medicine, Korea; 2 Department of Pediatrics,University of Ulsan, College of Medicine, Children’s Hospital, Asan Medical Center Seoul, Korea

**Keywords:** Congenital lipoid adrenal hyperplasia, twin

## 

Congenital lipoid adrenal hyperplasia (CLAH) is the most severe form of congenital adrenal hyperplasia that caused by mutations in the steroidogenic acute regulatory protien (*StAR*). The mutations in *StAR* gene resulted in failure of the transport cholesterol into mitochondria for steroidogenesis in adrenal gland.

Twin sisters (A, B) were born on 36^+2^ gestational week premature to nonrelated parent. Both patient A and B were phenotypic female with normal 46, XX genotype. They had symptoms as hyperpigmentation, slightly elevated potassium level and lower level of sodium without severe adrenal insufficiency symptoms. Laboratory finding reveals normal 17 hydroxyprogesterone level, elevated ACTH (A: 4379.2 pg/ml, B: 11616.1), high plasma renin activity (A: 49.02 ng/ml/hr, B: 52.7ng ml/hr). However, the level of plasma cortisol before treatment were normal (7.11 µg/dL) in patient A, but low (1.5 µg/dL) in patient B.

Patient A was readmitted with adrenal insufficiency symptoms at 38 days of age with concomitant infection which was suggestive of CLAH and prompted us to process a gene analysis and treatment was started in both. The results of gene analysis of *StAR* in twin revealed same heterozygous conditions for the c.544C>T (Arg182Cys) in exon 5 and c.722C>T (Gln258*) in exon 7.

We report a case of congenital lipoid adrenal hyperplasia showed different cortisol level in genotypic female twin with same *StAR* gene mutations.

